# Pushing back the limits of Raman imaging by coupling super-resolution and chemometrics for aerosols characterization

**DOI:** 10.1038/srep12303

**Published:** 2015-07-23

**Authors:** Marc Offroy, Myriam Moreau, Sophie Sobanska, Peyman Milanfar, Ludovic Duponchel

**Affiliations:** 1 LASIR CNRS UMR 8516, Université Lille 1, Sciences et Technologies, 59655 Villeneuve d’Ascq Cedex, France; 2 Department of Electrical Engineering, University of California at Santa Cruz, Santa Cruz, CA 95064 USA

## Abstract

The increasing interest in nanoscience in many research fields like physics, chemistry, and biology, including the environmental fate of the produced nano-objects, requires instrumental improvements to address the sub-micrometric analysis challenges. The originality of our approach is to use both the super-resolution concept and multivariate curve resolution (MCR-ALS) algorithm in confocal Raman imaging to surmount its instrumental limits and to characterize chemical components of atmospheric aerosols at the level of the individual particles. We demonstrate the possibility to go beyond the diffraction limit with this algorithmic approach. Indeed, the spatial resolution is improved by 65% to achieve 200 nm for the considered far-field spectrophotometer. A multivariate curve resolution method is then coupled with super-resolution in order to explore the heterogeneous structure of submicron particles for describing physical and chemical processes that may occur in the atmosphere. The proposed methodology provides new tools for sub-micron characterization of heterogeneous samples using far-field (i.e. conventional) Raman imaging spectrometer.

Spectroscopic imaging is a powerful technique for visualizing the spatial distribution and spectral information of complex samples^1^ (Fig. 1). Micro-Raman imaging instruments use a confocal microscope coupled to a spectrometer with a data collection system. The microscope focuses and collects the photons (scattered or non-absorbed) from a specific area of the analyzed sample. A motorized stage is adapted on the microscope enabling movement in the three directions of the space of the sample surface. Hyperspectral imaging consists in isolating the radiation spectrum associated with a specific vibration mode and then, the spatial distribution for a particular chemical component in the considered sample can be deduced from signal integration^1^.

Although hyperspectral images are powerful analytical measurements, far-field imaging spectrometers face restrictions on spatial resolution, especially when micro sized samples are considered. The spatial resolution limit is first and foremost dictated by the diffraction limit, which is often considered around one micron when a visible laser is used. Instrumental solution such as near—field spectroscopy was then developed to push the spatial resolution limit^2^ and successfully applied for the characterization of nanoparticles^3^. However, these approaches are often complex to apply from an instrumental point of view^2^ and are often not sensitive enough to characterize all species within sub-micrometric objects. Moreover, the small number of collected photons induces generally a low signal to noise ratio. In the 1980s, the signal processing community showed the possibility to overcome the limitations of optical systems. Thus, the image processing algorithm called super-resolution was born^4^,^5^. The main advantage of such signal processing approach is that it gives us the opportunity to keep our conventional far field Raman spectrometer as well as extend its spatial resolution. The main idea of super-resolution is the fusion of several low-resolution images of the same object observed from “different angles” in order to obtain one higher-resolution image^6^. The first results of a super-resolution algorithm were published by R. Y. Tsai and T. S. Huang^4^. Their main idea was to find a system of equations between the Discrete Fourier Transform (DFT) coefficients from low-resolution images and the high-resolution image sampling with the Continuous Fourier Transform (CFT). The system had a solution deduced from the inverse DFT in the frequency domain. For more than twenty years, different algorithmic approaches have been developed in the frequency or the multiple sampling domains^7^, including Bayesian theory^8^. These recent methods take into account the instrumental response and the “scene” movement to estimate a high-resolution image.

Our previous super-resolution works have demonstrated that it was possible to increase by about 30% the spatial resolution in Mid-infrared (MIR) and Near-infrared (NIR) spectroscopic imaging using a focal plane array detector on pharmaceutical samples^9^,^10^. It was more spectacular to demonstrate the spatial resolution increase at the SOLEIL French synchrotron facility for the analysis of a single HeLa cancer cell with MIR imaging^11^. In the present work, we explore the super-resolution concept coupled with chemometrics for the characterization of micron and sub-micron industrial particles with Raman imaging.

The first reason is to overcome the 1 μm spatial resolution usually considered as optimal for Raman imaging experiments in the visible range. Indeed due to the photon wavelength, the diffraction limit prevents observing details about micron or sub-micron sized particles. The second reason is to go further in the description of the chemical heterogeneity for individual atmospheric particles. Actually, atmospheric aerosols can be considered as heterogeneous entities resulting from atmospheric heterogeneous processes that occur at the particle size scale^12^. The impact of atmospheric aerosols on visibility and climate change as well as their toxicity depends on the size, the chemical composition, and the chemical mixing state (i.e. the spatial distribution of species within single particle) of the aerosols^13^,^14^,^15^. So far, a detailed knowledge about the aerosol composition is lacking for health risk assessment and to evaluate the environmental impact of aerosols.

The proposed chemometric strategy applied on aerosols samples will present a potential solution for obtaining molecular information on micron and sub-micron sized particles with *in situ* conditions; i.e. a better characterization of smaller particles with Raman imaging. The validation of the approach will be divided in two parts. First we will use an ideal sample called “target sample” to measure the intrinsic spatial resolution of the Raman instrument and to quantitatively evaluate the contribution of the super-resolution concept. In the second part, we will apply the super-resolution approach coupled with the multivariate curve resolution method (MCR-ALS) for the exploration of atmospheric particles.

## Results

### The intrinsic spatial resolution evaluation with a target sample

**Figure 2:**
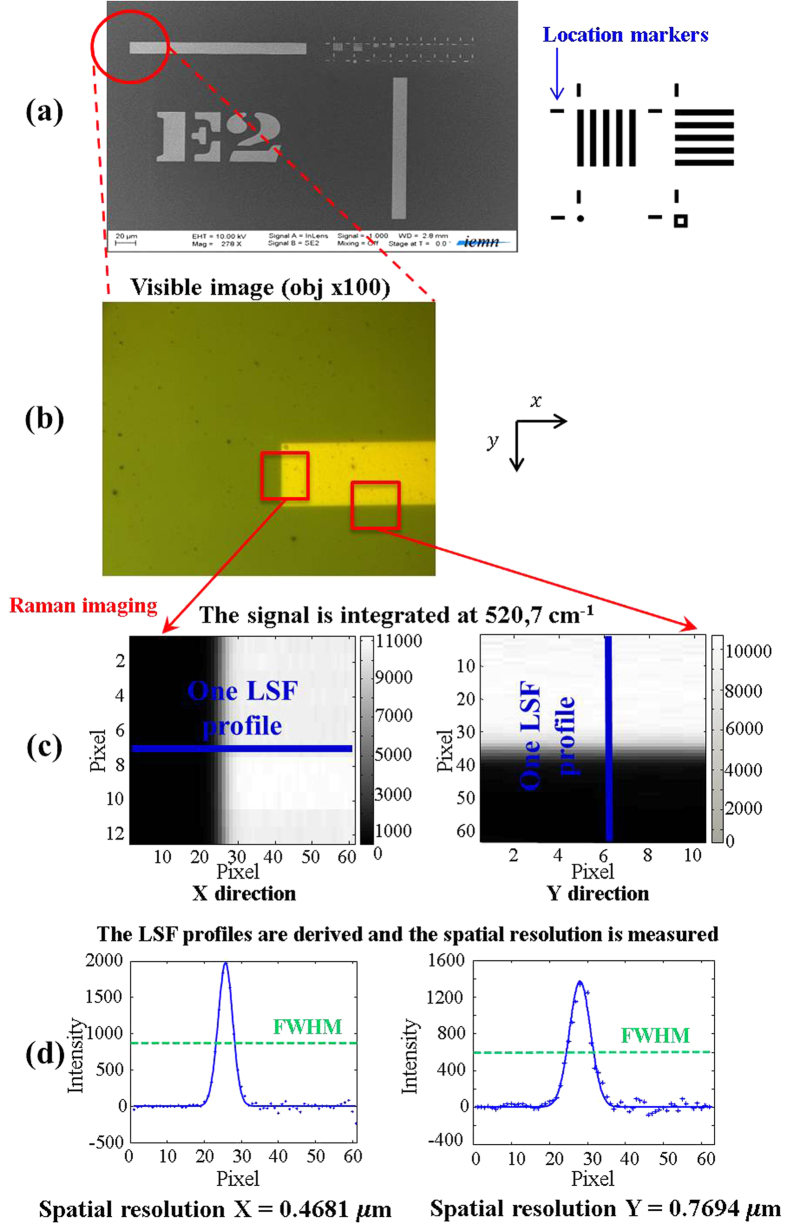
Spatial resolution method on the “target sample” with the “step-edge” criterion. (**a**) The target sample mask and the SEM general view with homothetic Au sub-micron patterns. (**b**) Optical image of location marker used as “step” to measure the spatial resolution. The relatively large width of the Au object (location marker) allows us to define two areas (red rectangles) in order to determine the spatial resolution in the x and y directions (**c**) After the Raman imaging on the areas, the typical silicon Raman band at 520.7 cm^−1^ is then integrated to generate maps. Considering the “step-edge” as an input signal presented to the optical system, the measured signal along perpendicular direction to the edge is its Line Spread Function (LSF) with respect to position (Blue lines). Due to the large analysis area, eight LSF profiles are measured (**d**) The LSF profiles are derived following the x and y directions in order to give associated Point Spread Function (PSF) profiles. In fact, the PSF is the response of the system to a point source observation. Each corresponding PSF function is then used as a measure of the spatial resolution by calculating the Full Width at Half Maximum (FWHM) with an associated standard deviation.

The “target sample” is considered as an ideal sample because it is spatially and chemically well defined. Electron lithography has been used to draw submicron Au patterns on a Si wafer surface^16^. In Fig. 2a, the Scanning Electron Microscopy (SEM) image presents a general view of the obtained Si target with submicron Au patterns. This sample structure was inspired by the Microscopy Resolution Test Chart (NBS 101A). It consists of five bars regularly spaced horizontally and vertically (Fig. 2a). The distance between two bars corresponds to the width of a bar. This five bars mask is replicated in a homothetic way in order to observe widths from 1 μm to 62.5 nm. Markers are also added to locate the different patterns when they become too small and almost invisible with a visible imaging system. The selected material was a Silicon wafer. Indeed it was a perfect candidate due to its good Raman scattering properties. Silicon gives a localized and intense spectral contribution at 520.7 cm^−1^ while no Raman scattering is observed for gold patterns. We have created this sample for two objectives. The first one was to evaluate the intrinsic spatial resolution along x and y directions (of the sample plane) with a quantitative criterion for different instrumental setups. The second objective was to estimate the new spatial resolution for the same instrumental setups after applying the super-resolution algorithm. The purpose was also to select experimental conditions for which the spatial resolution was the best before and after super-resolution process. The “step-edge” method^9^,^10^ is considered well suited to evaluate the spatial resolution of imaging systems. In this work, we have used a location marker from the Silicon wafer to measure the spatial resolution before and after applying the super-resolution algorithm (Fig. 2b–d).

In confocal systems, pinholes have a direct effet on spatial resolution. That is why four experiments with different pinhole sizes were performed to measure the intrinsic spatial resolution of the Raman instrument. The fifth experiment has been setup in order to observe the influence of the anti-vibration control system on the optical table (The instrument used is described in “Methods”). The spatial resolution was measured with the “step-edge” criterion on a location marker from the Si wafer. The mapping size was 10 × 60 pixels and 60 × 10 pixels in x and y direction respectively, with a step size of 100 nm. The spatial-resolution calculations have been done on eight LSF derivative profiles for each mapping. Table 1 gives the results of the intrinsic spatial resolution evaluation on the Raman instrument for different experimental conditions. First, we notice that the spatial resolution is significantly different in the x and y directions when the pinhole size is between 300 μm and 1000 μm (Experiment #1 to #3). This is explained by the fact that the Raman instrument is not in a real confocal mode in these conditions. Only a 200 μm pinhole gives the same spatial resolution in each direction. The experiments #4 and #5 show fairly equal spatial resolution, demonstrating that an active anti-vibration system does not change the intrinsic spatial resolution for conventional Raman measurements. Consequently, the intrinsic spatial resolution can be considered around 0.59 μm in each direction. Moreover, the calculations of the standard deviation confirm the excellent repeatability of the “step-edge” criterion. Indeed in confocal conditions, the standard deviation does not exceed 20 nm.

It is also interesting to note that the intrinsic spatial resolution estimated experimentally is much worse than the one we can obtain theoretically with the diffraction limit formula for a confocal system (Eq. 1).
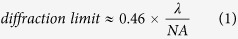
Indeed for the 532 nm wavelength laser and a × 100 objective (NA = 0.9) it would predict a spatial resolution of 272 nm. This first result provides strong evidence that the spatial resolution must always be estimated experimentally in order to have an unbiased vision of the instrumental performance.

### Characterization of the improved spatial resolution when using the concept of super-resolution

The spatial resolution of the super-resolved image was measured following the previous methodology. We have then demonstrated the power of this approach by analyzing a particular pattern from the target sample. As the intrinsic spatial resolution of the system was around 0.59 μm, it seemed interesting to use the super-resolution approach on the pattern with corresponding widths i.e. 0.5 μm. We then focused on the pattern with 5 vertical lines.

In order to apply super-resolution, a set of low-resolution images was acquired by inducing a sub-pixel shift (lower than the pixel size) in the x and y directions between them. In this way, 100 images with a pixel size of 1 μm, shifted from 100 nm from each other in x and y directions were acquired. All data cubes were integrated at 520.7 cm^−1^ to obtain 100 low-resolution images. The super-resolution algorithm was then used to generate a high-resolution image. Table 2 presents the spatial resolution of the super-resolved image calculated with the “step-edge” criterion. As we can see, the new spatial resolution is always better than the intrinsic spatial resolution. However, there is still a significant difference between the x and y directions for a pinhole size from 300 μm to 1000 μm. Only a 200 μm pinhole size provides an equivalent spatial resolution in each direction. In this condition, the spatial resolution is around 200 nm. It corresponds to an improvement of at least 65% compared with the intrinsic spatial resolution in the same experimental conditions. When considering our previous results of super-resolution in near infrared and mid-infrared spectroscopic imaging, we can state that these new results are by all means the most spectacular.

**Figure 3:**
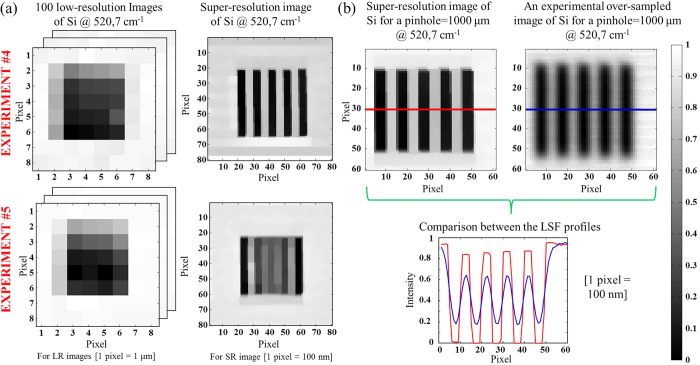
Super-resolution concept applied on the target sample. (**a**) We present images of a 5-bars pattern from the target sample (bar width = 500 nm) before and after using super-resolution for the experiment #4 and #5. Low-resolution images (LR) have 8 × 8 pixels (1 μm per pixel) whereas super-resolution images (SR) have 80 × 80 pixels (100 nm per pixel). The super-resolution images were obtained with the fusion of 100 low-resolution chemical images of Si shifted from 100 nm from each other in x and y directions. (**b**) The super-resolution image from experiment #1 is compared with the integrated image directly obtained with the same pixel density and experimental conditions. Red and blue lines represent the LSF profiles.

We can also notice that the resolution values of experiments #5 are not indicated in Table 2. Indeed the super-resolution algorithm failed to find a high-resolution image in these conditions (Fig. 3a). As one can see, experiment #5 does not allow us to retrieve the pattern while experiment #4 does. This fact indicates that even using a piezo stage is not enough when applying super-resolution. Indeed, the anti-vibration system seems to be necessary to obtain the highest reproducibility in position. When for the first time we have presented super-resolution results for other spectroscopic techniques, people thought the concept was just over-sampling. That is why in Fig. 3b we have compared the super-resolution image from experiment #1 with the integrated image directly obtained with the same pixel density. The new mapping step over the 5-bars pattern is 100 nm with a 1000 μm pinhole size and activated anti-vibration system. It is obvious that the spatial resolution and the contrast are significantly improved when super-resolution algorithm is used. It is also observed in the associated LSF profiles.

This first part focused on the target sample was essential to prove the concept and determine optimized environmental and instrumental conditions in order to achieve the best spatial resolution when using the super-resolution concept. For the following aerosol study, the experimental conditions were fixed to a 200 μm pinhole and an activated anti-vibration system.

### Spectral and Spatial Aerosols characterizations

The aim of this part is to characterize aerosol particles in order to identify all the pure components by Multivariate Curve Resolution and Alternative Least Squares^17^,^18^,^19^ (MCR-ALS) in the collected sample. The information in each specific compound set of maps is directly used with a super-resolution post-processing algorithm (see the Methods section). The sample of aerosol was collected in the courtyard of a lead-acid battery-recycling factory located in Toulouse, France. The particles were collected by impaction on silver sheets using a cascade impactor (Dekati Ltd., Finland) operating at a flow rate of 10 l⁄min. The impactor setup consists in three successive stages with aerodynamic cut-off diameters (AD) which correspond to the size segregation of 2.5 μm < AD < 1 μm (stage 1), 1 μm < AD < 0.1 μm (stage 2) and AD < 0.1 μm (stage 3). The collection time was one hour to ensure the particle dispersion on the collecting substrate. In this study, only stage 2 was examined to demonstrate the feasibility of the analytical methodology considering sub-micron sized particles.

**Figure 4:**
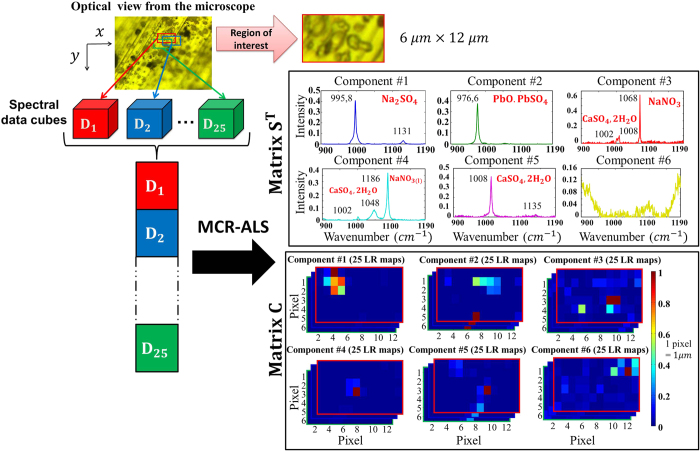
Multivariate Curve Resolution and alternating Least Squares (MCR-ALS) analysis of the image multiset from the atmospheric particles. The optical view from the microscope shows the selected region of interest (6 × 12 μm). Then, the 25 data cubes were acquired with the Raman instrument. After removing the fluorescent effect on spectra, the MCR-ALS was performed on a column-wise augmented matrix. We obtained the spectral matrix S^T^ which contained the 6 pure components and concentration matrix C which contained 25 chemical map for each components.

As the first step, we explored the sample of collected aerosols in order to select a region of interest (Fig. 4). Then, we optimized the experimental conditions of acquisition for this area of 6 × 12 μm to obtain the best signal-to-noise ratio. The acquisition time was thereby fixed to 40 min per data cube. We produced 25 data cubes shifted from multiple of 200 nm in the x and y directions. Pre-processing by Weighted Least Squares^20^,^21^ (WLS) was used on the raw spectra to remove the fluorescent effect. MCR-ALS was performed on the 25 sub-matrices **D** structured as a column-wise augmented matrix, since the spectral direction was common to all data cubes (from 900 cm^−1^ to 1190 cm^−1^). Therefore the multiset structure contained the 1800 WLS-corrected spectra (25 × 6 × 12). After detecting a rank of six for **D** matrix (i.e. six pure contributions) by Singular Value Decomposition^18^ (SVD), initial estimates of pure spectra were obtained by SIMPLISMA^18^ applied to the full multiset of low-resolution images. The non-negativity constraint^18^ was used in the resolution analysis on the concentration profiles and spectra. The normalization constraint^18^ was also applied in **S**^T^.

Figure 4 shows the results of the six extracted chemical compounds. For each contribution the 25 low-resolution images are generated from the refolded concentration matrix denoted **C**. The pixel size is 1 μm for each low-resolution image. The lack of fit is 9.99% and the explained variance is 99.00% which is satisfactory for the considered aerosol Raman images. On the same figure, one can observe interesting pure spectral profiles. First, the contribution #6 is due to uncorrected fluorescence effect on the boundary of the spectral domain. Contributions #1 to #5 are pure chemical compounds or mixtures. The identification of these five contributions has been made based on the literature^22^,^23^,^24^. The particles collected are mainly composed of sea salt derived particles (i.e. Na_2_SO_4_ and CaSO_4_.2H_2_O), or reacted particles (i.e. NaNO_3_) in aggregation with submicronic metal-rich particles (PbO.PbSO_4_). In addition, internal mixing of sea salt derived particles is observed (i.e. Na_2_SO_4_ and CaSO_4_.2H_2_O). The co-crystallization during the sea salt droplets evaporation in the atmosphere explains the formation of mixed solid particles. The distribution maps show a rough localization of the pure chemical compounds. They are blurred and present low spatial resolution as expected from the large 1 μm pixel size in the original maps.

**Figure 5:**
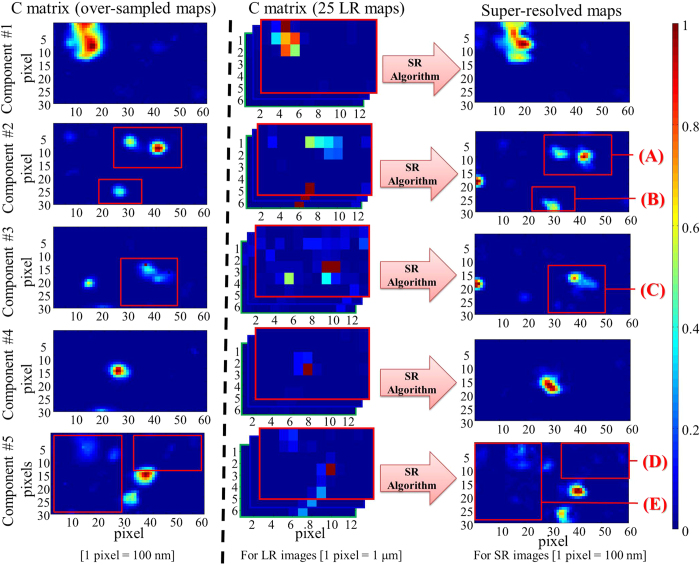
Comparison of the distribution maps obtained from MCR-ALS analysis of over-sampled images (left plots), MCR-ALS multiset image analysis of the low-resolution images (center plots), and the super-resolution images (right plots). The component #1 is much better described on the super-resolution image. The edges and the shape of the aerosol aggregates are better defined. It is also observed for area (**A**) and (**B**) on the component #2. This is even more evident on the component #4. A single particle is detected on the over-sampled map while two stuck particles seem to be observed on the super-resolution image. Some new aggregates can even be observed on the component #3 (**C**) or on the component #5 (**D**) and (**E**) for the super-resolution images that are impossible to observe on the over-sampled images.

At this stage, each chemical contribution is represented by 25 distribution maps, shifted slightly from each other. To obtain a single distribution map per contribution with higher spatial resolution, the super-resolution post-processing step was applied separately to each set of 25 low-resolution distribution maps. In other words, a super-resolution map for each contribution is obtained from the fusion of 25 maps (6 × 12 pixels) with a pixel size of 1 μm and shifted with a motion step of 200 nm between them in the x and y directions. These new super-resolution maps (60 × 30 pixels) have then a pixel size equal to 200 nm. As for the target sample analysis, we present in Fig. 5 images of the five components obtained from a single data block with an oversampling step of 200 nm on the same aerosol sample area. The map of each chemical compound was extracted with the same MCR-ALS and WLS procedures previously used on the 25 data blocks. In this way, the over-sampled and super-resolution images have the same pixel density for each pure contribution. Considering the results, one can notice directly the improvement in spatial resolution and contrast on the super-resolution results compared with over-sampling procedure.

These results clearly evidence that the MCR-ALS methodology coupled with the super-resolution concept lead to richer information on physicochemical description of single particles collected in industrial area than that obtained with the classical Raman imaging approach. In particular, the significant improvement of the species distribution maps reveal aggregation of sub-micronic particles with a single composition whereas classical Raman imaging show a unique particle with a micronic size composed of several species in internal mixing.

## Discussion

Our present work has demonstrated the possibility to surmount instrumental limits in Raman imaging. The first step of our approach was to measure the intrinsic spatial resolution on the Raman instrument with an ideal sample. It has been shown that the spatial resolution was particularly dependent on the experimental conditions. Indeed, it was surprising to see the importance to work with an anti-vibration system in order to obtain the best reproducibility in position. As we have seen, the super-resolution algorithm was not able to converge when it was not activated. Considering the analysis of the target sample, it has been shown first that even the intrinsic spatial resolution has to be determined experimentally in order to have an unbiased vision of the analytical performance. Using the super-resolution algorithm, it was possible to improve the spatial resolution by 65% considering a pinhole size of 200 μm and an activated anti vibration system. This spatial resolution around 200 nm in the x and y directions is significantly better than the diffraction limit on an ideal confocal system, which is a rather spectacular result. This gain is far from negligible when submicron samples are considered.

The second part of the study was to develop a new strategy to analyze sub-micron industrial aerosols with Raman imaging. That consisted in practice of a proper design of the acquisition of a series of hyperspectral images with a small motion step among them, as small as the pixel size desired, combined with suitable chemometric tools. The MCR-ALS multiset image analysis coupled with super-resolution post-processing was then an efficient way to resolve the internal structured of the segregated particles (0.1–1 μm size range) collected in an industrial area. The MCR-ALS step was able to reveal the molecular composition of individual particles. The successful characterization of heterogeneous structures of submicron particles is clearly evident with the presented methodology. The MCR-ALS algorithm coupled with super-resolution can be relevant to understanding the heterogeneous structure of submicron aerosols for describing physical and chemical processes that may occur in the atmosphere. We are convinced that the proposed methodology will provide new trends for a sub-micron characterization of heterogeneous samples using far-field Raman imaging.

## Methods

### Instrumentation

All Raman hyperspectral images were acquired with the LabRAM HR confocal scanning spectrometer manufactured by Horiba Jobin Yvon Scientific Company. The spectrometer is coupled confocally with an Olympus BX 40 high-stability microscope equipped with × 100 objective (NA = 0.9). This instrument is equipped with a 1200 grooves/mm holographic grating that enables a spectral resolution of 0.5 cm^−1^. A liquid nitrogen-cooled CCD detector is used in the spectrometer. Raman backscattering is obtained with a 532 nm excitation wavelength (25 mW) supplied by a solid-state laser. The samples are placed under the microscope on a XYZ piezo motorized stage from PI (Physik Instrument). This stage offers movement up to 100 μm in the three space directions with a step that can achieve the nanometer (if necessary). The z direction of the table is not changed during the super-resolution experiments. It is only fixed at the beginning in order to focus on the surface of the sample. All the instrumental setup is placed on an optical table with a vibration control system ST-UT2 from Newport Corporation, USA. It uses pneumatic cells inside the table legs to detect and to damp the low-frequency vibrations due to objects in the environment (like people walking, building equipment, passing automobiles, etc…). This device can be activated when required for the experiment.

### The Target Sample fabrication by lithography

**Figure 6:**
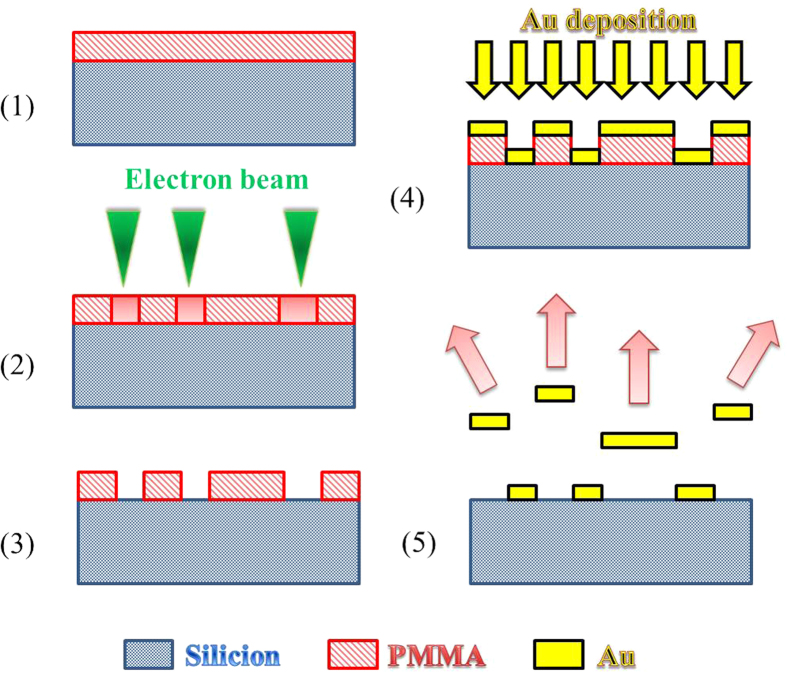
Sample preparation with electron lithography. Consecutive steps: (**a**) PMMA polymer coated onto a Si sample. (**b**) Electron beam exposure. (**c**) Development phase. (**d**) Au metal deposit by evaporation. (**e**) Lift-off.

The “target sample” was realized by Electron lithography (Fig. 6). First a poly methyl methacrylate (PMMA) electron sensitive resist solution was spin-coated onto Si sample and baked to leave a hardened thin-film on the surface (around 500 nm). An electron beam has scanned the sample following the five bars mask and caused PMMA chain-scissions. These fragmented polymer chains were then dissolved by a developer solution. A 50 nm thickness of gold was then deposited by evaporation over the entire sample surface. The final stage, called lift-off was the immersion of the sample in acetone to remove any remaining traces of PMMA and finally reveal the gold patterns.

### The super-resolution concept

Super-resolution is a discipline of the signal processing research field. It is defined by the use of image processing methods in order to overcome limitations of optical systems. The concept of the super-resolution algorithm is the fusion of several low-resolution images of the same sample observed from slightly shifted point of view to generate one image with higher spatial resolution^5^,^6^.

**Figure 7:**
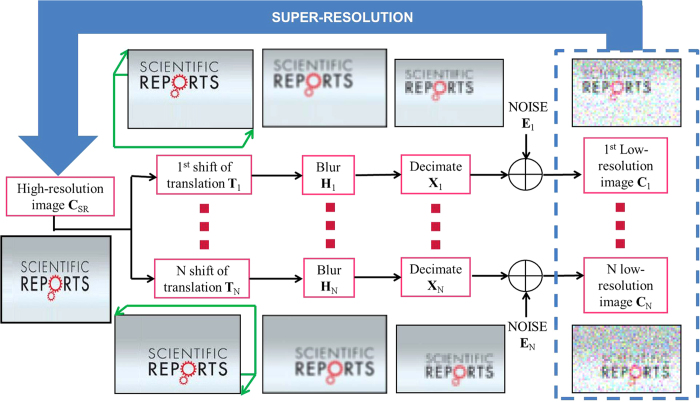
Representation of the analytical model used in the super-resolution concept. A low-resolution image is a noisy, decimated, blurred and warped version of the original high-resolution image. The concept of the super-resolution is to find a high-resolution image with the fusion of the low-resolution images with the knowledge of the noise, the decimate, the blur and the shift of the “scene”.

The *N* low-resolution maps are denoted 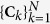. These low-resolution images (low pixel density) are defined by *M*_1_ × *M*_2_ pixels. They can be considered as worse and different representations of a single map of much-higher resolution **C**_*SR*_ defined by *L*_1 _× *L*_2_ pixels, where *L*_1_ > *M*_1_ and *L*_2_ > *M*_2_ for 1 ≤ *k* ≤ *N*. In our experiment with the “target sample”, 100 shifted maps are considered (*N* = 100). The parameters *L*_1_ and *L*_2_ are user-defined that always satisfy the following rule of thumb:
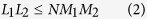
Let *L*_1_ = *kM*_1_ and *L*_2_ = *kM*_2_. We must select the highest *k*-value to satisfy Eq. 2 taking into account *N* = 100 and *M*_1_ = *M*_2_ = 8 in our case. Thus the *k* value was fixed to 10 (i.e. *L*_1_ = *L*_2_ = 80). The super-resolution concept considers that each low-resolution map **C**_*k*_ is the result of a particular geometric warping (in our case translation in x and y directions), linear space-invariant blurring and uniform rational decimation (subsampling) performed on the ideal high-resolution map **C**_*SR*_. In general we also consider that the low-resolution images are corrupted with an additive Gaussian noise. It is thus possible to propose an analytical model (Eq. 3) in order to express the steps previously described:
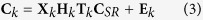
The effect of each operations considered in the analytical model is displayed in Fig. 7. The matrix **T**_*k*_ sized [*L*_1_*L*_2_ × *L*_1_*L*_2_] corresponds to the geometric translation between the **C**_*SR*_ map and **C**_*k*_. The matrix **H**_*k*_ sized [*L*_1_*L*_2_ × *L*_1_*L*_2_] is the blur matrix defined by the optical system Point Spread Function (PSF). The matrix **X**_*k*_ sized [*M*_1_*M*_2_ × *L*_1_*L*_2_] is the decimation resulting in **C**_*k*_. It corresponds to the reduction of the number of observed pixels in the measured image. The additive Gaussian noise observed in the *k*-th map is described with **E**_*k*_.

Considering a generalization of this model applied to all *N* available low-resolution maps, we have the system of equations below:
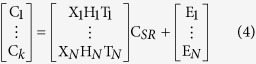
From that moment, super-resolution becomes an inverse problem since **C**_*SR*_ must be found from the set of measured low-resolution maps 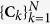 and the estimation of all **X**_*k*_, **H**_*k*_ and **T**_*k*_ matrices (*k* = 1, …, *N*). For our spectroscopic experiment, **T**_*k*_ matrices are just defined by the shifts between low-resolution maps 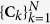, with respect to one of them taken as a reference. These shifts are translations induced by the XY piezo stage which are multiple of 100 nm between two map acquisitions. The main idea of the super-resolution concept is that these shifts have to be lower than the pixel size of the low-resolution image which is the case here. Moreover we consider **H**_*k*_ as constant because all low-resolution images are obtained with the same optical system. We also make the assumption that the PSF remains constant all over the time experiment. In general, this matrix is estimated experimentally but even a rough guess of it, like a Gaussian filter can be used. The matrix **X**_*k*_ depends only on the decimation ratio between the high-resolution image and the low-resolution images i.e. the ratio between the number of pixels of the two. Ultimately, in light of all these considerations, it is now possible to retrieve the high-resolution map **C**_*SR*_ with a classical restoration method such as Maximum A Posteriori (MAP) estimation^8^. This methodology comes from Bayesian statistics. MAP is particularly well suited for solving inverse problem. It is closely related to Maximum Likelihood (ML) method, but employs an optimization procedure, which incorporate prior information. A regularization term is necessary in order to find a stable solution of **C**_*SR*_. We have here what we call an ill-posed problem because of the high dimension of the equation system (Eq. 4) and inherent numerical instability of such inverse problem. The algorithm called “Norm L_1_ + Bilateral Total Variation” was then chosen to retrieve the super-resolved maps on the pure aerosols constituents^5^,^6^,^8^.

### Spectral data pre-processing

**Figure 8:**
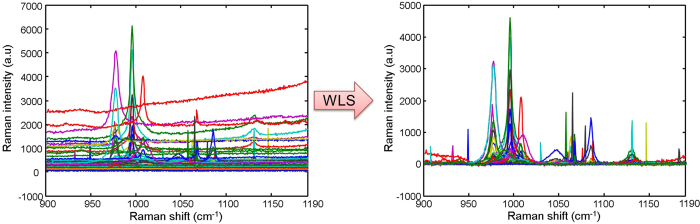
Spectral data pre-processing. (**a**) Spectra from the aerosols’ region of interest (12 × 6 pixels). (**b**) Spectra are corrected with the Weighted Least Squares (WLS).

The intense background associated with fluorescence contributions is a common problem in Raman imaging. Indeed, Raman spectra have intense and irregularity shaped baselines changing from pixel to pixel. The raw spectra need to be corrected in order to extract the unbiased and real chemical information. The weighted Least-Squares (WLS), also called Asymmetric Weighted Least-Squares (AsLS) removes the fluorescent contributions^20^ (Fig. 8). This pre-process algorithm was proposed originally by Eilers *et al.* to subtract baseline shifts in chromatography. This method is based on a recursive local fitting of the entire spectrum with a baseline obtained by using a Whittaker smoother^21^.

### Multivariate curve resolution of hyperspectral Images

**Figure 9:**
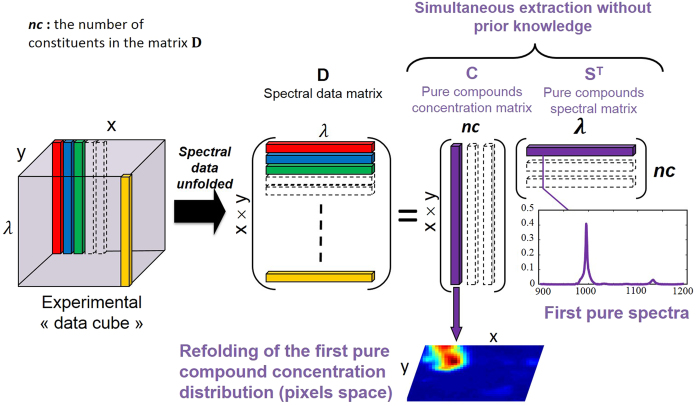
The Multivariate Curve Resolution in hyperspectral imaging. The experimental “data cube” is unfolded to obtain the spectral data matrix noted **D**. A multivariate curve resolution is applied to obtain simultaneously the pure compounds concentration and spectral matrices respectively **C** and **S**^**T**^. The advantage of this methodology is to extract the spatial and spectral information without prior knowledge like the wavelength selectivity.

In hyperspectral Raman imaging, data acquired usually correspond to a data cube (three dimensions i.e. *x Pixels* × *y Pixels* × *λ wavelengh*). First, the data cube is unfolded into a matrix **D** (two dimensions) with *x* × *y* rows and *λ* columns. The goal of hyperspectral image resolution is the decomposition of the raw image spectra **D** into the pure distribution maps **C** and pure spectra of all the constituents **S**^T^ present in the aerosol sample analyzed (Fig. 9).

This decomposition is carried out according to the bilinear model presented in Eq. 5^17–19^:
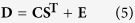
The matrix **E** corresponds to the experimental error contained in the raw measurement. Multivariate Curve Resolution**-**Alternating Least Squares (MCR-ALS) is an iterative curve resolution method used to recover the underlying spectroscopic bilinear model in Eq. 5. R. Tauler and A. De Juan^17^,^18^ have developed this method. After the MCR analysis of the data cube, the pure distribution maps of all constituents can be obtained by folding back the concentration profiles in **C** to recover the original 2D spatial structure of a classical image^19^,^25^,^26^,^27^. The MCR-ALS algorithm is based on the following steps:

1. Determination of the number of constituents in the matrix **D** by Singular Value Decomposition (SVD)^18^.
2. The generation of initial estimates **S**^T^. The SIMPLISMA algorithm is used for the selection of the purest pixel spectra in image analysis^18^.
3. Alternating Least-Squares (ALS) calculation of **C** and **S**^T^ under constraints.

The ALS step involves the operations **C** = **DS**(**S**^T^**S**)^−1^ and **S**^T^ = (**C**^T^**C**)^−1^**CD** respectively. The end of the iterative process takes place when the product of the resolved concentration profiles and spectra reproduce the original data **D** without significant variation between consecutive cycles. The parameter used to measure the fit quality of the MCR-ALS model is the explained variance (*r*^2^) and the lack of fit (*lof* (%)) defined as follows:
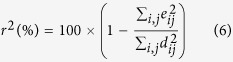

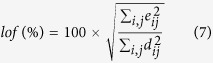
Where *e*_*ij*_ are the elements of the residual matrix **E** and *d*_*ij*_ are the elements of the dataset **D**. The subindexes *i* and *j* refer to the pixel and wavenumber, respectively. During the MCR-ALS steps, the algorithm allows introducing prior knowledge about images by the use of constraints on **C** and **S**^T^ profiles. The use of constraints in the resolution process helps to decrease rotation ambiguity effects in the final results obtained i.e. lower the total number of feasible solutions. The most commonly used constraint in hyperspectral image analysis is non-negativity in the concentration and the spectral directions. In our case, this constraint is applied because the concentration of the constituents in the image and the spectroscopic readings of the WLS-corrected Raman spectra are positive. Normalization of pure spectra in **S**^T^ is also a common constraint used to avoid scaling fluctuations in the profiles during optimization.

The simultaneous analysis of several images can be an asset to understand the analyzed sample. Multiset structures or augmented data matrices **D** contained in this case different sub-matrices **D**_i_. Image multiset analysis is clearly based on the bilinear model of the the Beer-Lambert law (Eq. 5). The pixel spectra of all images are organized in a data table **D**, where a block (or sub-matrix) belongs to a particular image. MCR-ALS for multiset image analysis follows the same steps mentioned above for single image analysis, but benefits from the complementary information contained in the different images to enhance the quality and precision of the final results. Thus, the decomposition **D** = **CS**^T^ + **E** provides a single matrix **S**^T^ of pure spectra, valid for each images sets, and a matrix **C**, formed by as many submatrices as images in the multiset structure. The profiles in each of these sub-matrices can be refolded conveniently to recover the related distribution maps of each image.

### The super-resolution post-processing strategy on aerosols

The super-resolution algorithm is used on each set of the distribution maps obtained from the MCR-ALS multiset analysis of the low-pixel-density aerosol images i.e. 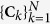 represent a particular image constituent from MCR analysis, where each map is defined by *M*_1_ × *M*_2_ pixels. Considering the 25 shifted maps in our experiment, *N* = 25, *M*_1_ = 12, *M*_2_ = 6, *L*_1_ = 60 and *L*_2_ = 30. These user-defined parameters always satisfy the rule of thumb in Eq. 2. The number of low-resolution image (25) and the motion step (0.2 μm) differ from the super-resolution post-processing of the target sample. Compared with the study of the pattern, we reduced the number of data cubes to avoid sample degradation over time and also to reduce the total acquisition time.

## Additional Information

**How to cite this article**: Offroy, M. *et al.* Pushing back the limits of Raman imaging by coupling super-resolution and chemometrics for aerosols characterization. *Sci. Rep.* **5**, 12303; doi: 10.1038/srep12303 (2015).

## Figures and Tables

**Figure 1:**
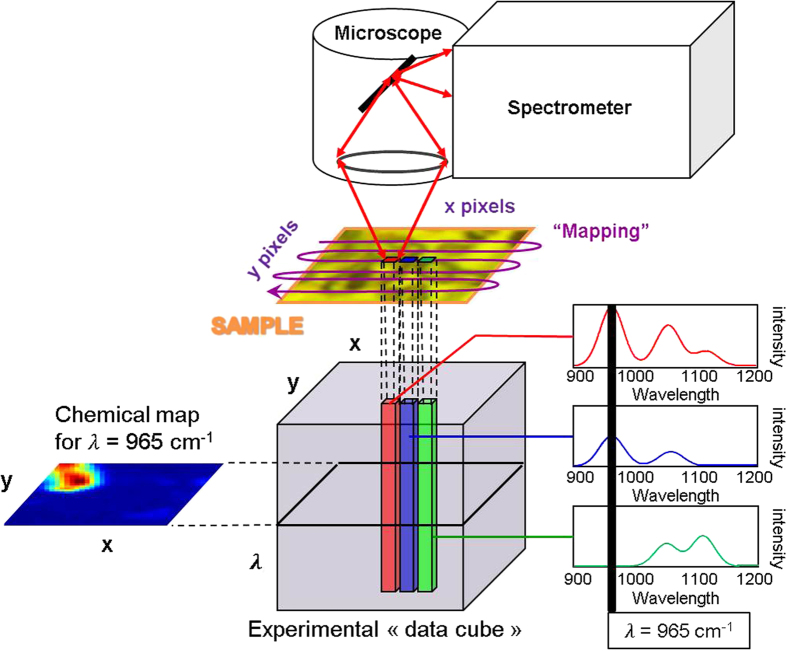
Hyperspectral imaging. A “mapping” in the x and y directions is used to acquire the experimental data cube (x pixels × y pixels × λ). As a consequence, each pixel has a spectrum. If we know, for example, that the λ = 965 cm^−1^ is a specific wavelength of the compound of interest, it is then possible to integrate the signal for each pixel to generate its corresponding chemical map.

**Table 1:** **The intrinsic spatial resolution of the Raman instrument measured for different experimental settings**.

**Intrinsic spatial resolution**						
**Experiments**	**Pinhole size (μm)**	**Anti-vibration system state**	**Intrinsic spatial resolution along** ***X*****-direction (μm)**	**Standard deviation** ***σ***_**X**_ **(μm)**	**Intrinsic spatial resolution along** ***Y*****-direction (μm)**	**Standard deviation** ***σ***_**Y**_ **(μm)**
**#1**	1000	ON	0.47	0.01	0.77	0.01
**#2**	500	ON	0.46	0.02	0.78	0.02
**#3**	300	ON	0.52	0.01	0.66	0.02
**#4**	200	ON	0.59	0.02	0.56	0.01
**#5**	200	OFF	0.57	0.01	0.62	0.01

**Table 2:** **The spatial resolution of the instrument after applying a super-resolution algorithm for different settings**.

**Spatial resolution after using super-resolution**						
**Experiments**	**Pinhole size (μm)**	**Anti-vibration system state**	**New spatial resolution along** ***X*****-direction (μm)**	**Standard deviation** ***σ***_**X**_ **(μm)**	**New spatial resolution along** ***Y*****-direction (μm)**	**Standard deviation** ***σ***_**Y**_ **(μm)**
**#1**	1000	ON	0.18	0.01	0.26	0.02
**#2**	500	ON	0.19	0.01	0.33	0.01
**#3**	300	ON	0.20	0.02	0.25	0.01
**#4**	200	ON	0.19	0.01	0.22	0.01
**#5**	200	OFF	—	—	—	—
